# Structural Characterization of Polysaccharides in Waste Liquor Produced by Wet Decortication of Sesame Seeds

**DOI:** 10.3389/fnut.2022.940442

**Published:** 2022-06-13

**Authors:** Yao-Ran Li, Shuai Xu, Run-Yang Zhang, Ming-Xuan Yang, Hua-Min Liu, Xue-De Wang

**Affiliations:** ^1^College of Food Science and Technology, Henan University of Technology, Zhengzhou, China; ^2^College of Biosystems Engineering and Food Science, Zhejiang University, Hangzhou, China

**Keywords:** characterization, polysaccharide, waste liquor, wet decortication, sesame seeds

## Abstract

The wet decortication of sesame seeds produces wastewater containing diverse minerals and organic pollutants that could be valuable resources for the food industry. This investigation aimed to reclaim, purify, and characterize the polysaccharides contained in the waste liquor from the sesame decortication industry. The purified polysaccharide fractions were characterized using monosaccharide analysis, GPC (high-performance gel permeation chromatography), FT-IR (Fourier-transform infrared) spectroscopy, methylation analysis, 1D and 2D Nucleai Magnetic Resonance (NMR) analysis, and thermal analysis. Four fractions were found (SSP-1,-2,-3, -4), of which SSP-2 was proportionately the largest and most interesting. The backbone of SSP-2 is mainly composed of (1→2,4)-β-_D_-Xyl*p* residues with side chains connected to the O-4 position, with many T-β-_D_-Gal*p* and (1→5)-α-_L_-Ara*f* residues, and fewer (1→4)-α-_D_-Glc*p*, (1→2)-α-_L_-Rha*p*, T-α-_L_-Ara*f*, and (1→2)-β-_D_-Glc*p*A residues. An efficient method for removing the polysaccharides would simplify wastewater treatment while finding a use for them would benefit the sesame, food, and pharmaceutical industries.

## Introduction

Sesame (*Sesamum indicum* L.), primarily cultivated in Burma, Sudan, China, and India since ancient times, consisting 60% of the world’s total yield, is one of the most important annual oilseed crops (1, 2). With its high content of protein and oil, sesame is a nutritious food, widely consumed in sweet and savory dishes and confections (3, 4). Typically, sesame seeds are dehulled before being used as human food. The meal left from oil extraction is high in protein, indigestible fiber, and oxalic acid. It is used to a limited extent as animal feed and might, with further treatment, be a source of protein for human food products (5, 6).

Among the current dehulling methods, two of the most common approaches are dry decortication and wet decortication. Dry decortication entails simply heating the sesame seeds. It is environmentally friendly as it produces no waste liquor; however, the long time and high temperature of the roasting process may change the appearance and flavor of the kernels (7). Wet decortication involves the use of a strong alkali to remove the hulls. It is a conventional method that used to be widely adopted by the industry because the method can provide kernels with a more desirable appearance, taste, and yield than dry decortication. The use of lye, however, has significant disadvantages, such as its corrosive effect on equipment, the possible hazards of applying the hot (60°C) alkali, and the heat costs to keep the alkali at 60°C during the operation (7). The greatest drawback, however, is that a large volume of water is needed to wash off the lye, and the disposal of this waste lye liquor remains a common and serious problem confronting sesame producers all over the world (8).

Several studies have suggested that valuable nutrients can be reclaimed from the byproducts of sesame dehulling, and utilized in various ways (9). As for disposal of the wastewater, if this water could be further and efficiently processed to yield valuable resources, the benefits would be far-reaching. The waste lye liquor, one of the byproducts obtained during the process of wet decortication, is mainly composed of proteins and a range of polysaccharides, such as pectin, gums, celluloses, and hemicelluloses. Polysaccharides, very important bioactive macromolecules, have long been used as additives in healthy foods due to their various biological effects and in emulsions to improve texture, water retention, and stabilization (10). These molecules are proven to have antioxidative, hypolipidemic, and anti-tumor activities (11–13). The complex structures of polysaccharides, including the variety of monosaccharide components and the various linkage and configuration patterns, give them their diverse functional properties (14). To the best of our knowledge, the sugar composition and structural characterization of the sesame polysaccharides obtained from the waste liquor produced in the process of wet sesame dehulling have not been studied. We believe that these polysaccharides could be valuable resources for industry and that an efficient method for removing them would both benefit the sesame industry and simplify wastewater treatment.

Thus, the purpose of the present investigation aimed to develop an efficient, effective way to reclaim natural polysaccharides from the waste liquor produced by the wet decortication of sesame seeds. Polysaccharide fractions were reclaimed and purified, and the composition and structural features of the purified polysaccharide fractions were characterized using monosaccharide analysis, GPC (high-performance gel permeation chromatography), FT-IR (Fourier-transform infrared) spectroscopy, methylation analysis, 1D and 2D Nucleai Magnetic Resonance (NMR) analysis and thermal analysis.

## Materials and Methods

### Materials

Sesame seeds (*Sesamum indicum* L.) were obtained from the Sesame Research Center, Henan Academy of Agricultural Sciences. After sieving to remove impurities, the seeds were dried for 24 h at 40°C and then kept in a sealed container at 4°C until decortication. Chemicals required for the assays, including dextran standards and standard monosaccharides (_D_-glucose, _D_-galactose, _D_-mannose,_D_-xylose,_L_-rhamnose, _L_-arabinose, _D_-galacturonic acid, _D_-glucuronic acid) were purchased from Sigma Chemical Co. (St. Louis, MO, United States). DEAE Cellulose-52 was obtained from Solarbio Science and Technology Ltd. (Beijing, China). All other chemical reagents were acquired from Tianjin Chemical Reagents Co. Ltd. (Tianjin, China) of analytical grade.

### Sesame Wet Decortication Waste Liquor Collection

In brief, 500 g of sesame seeds were soaked in 3 L of 0.25% NaOH lye solution at 60°C for 5 min with stirring (500 rpm). The mixtures were passed through a 60-mesh sieve, and the sesame seeds were collected. After washing with rushing water to eliminate the residual lye, the seeds were rubbed for 5 min to decorticate the hulls. All hot-alkaline waste liquor was retained for further analysis.

### Reclamation of Crude Polysaccharide

Firstly, the collected waste liquor was filtered to remove impurities. The pH of the filtrate was adjusted to 5.5, and the solution was centrifuged at 4,000 rpm (Backman, Avanti J-25, United States) for 15 min to precipitate sesame protein. The supernatant was collected and concentrated by a rotary evaporator (RE-3000A, Yarong Technology and Science Inc., Shanghai, China) and then precipitated with three times 95% (v/v) ethanol; solutions were left to settle for 3 h. The insoluble contents were collected by filtering through double layers of filters; afterward, the precipitate was washed with 70% ethanol. The precipitate was then decolorized with AB-8 macroporous resin and deproteinized by the Sevag method (15). To remove the salt and small molecule impurities, it was dialyzed for 3 days against water (molecular weight cut-off Mw 3,500 Da) and lastly lyophilized to obtain the refined crude polysaccharide (SSP).

### Purification of the Polysaccharide Fractions

The crude SSP (500 mg/mL) was further dissolved in distilled water and separated on a DEAE cellulose-52 column (2.6 cm × 30 cm) by eluting with 0, 0.1, 0.2, 0.3, 0.5, and 0.7 M NaCl solution at a rate of 10 mL/tube (1.5 mL/min). The collected fraction was measured at 490 nm defining _D_-glucose as a standard by the phenol-sulfuric acid method (16). The four main fractions corresponding to the four concentrations of NaCl solution (0.1, 0.2, 0.3, and 0.5 M), were collected and named SSP-1, SSP-2, SSP-3, and SSP-4, respectively.

### Characterization

#### Monosaccharide Composition Analysis

A total of 5 mg of each polysaccharide sample was hydrolyzed by 3% H_2_SO_4_ at 105°C for 2.5 h. Samples were cooled to room temperature, then filtered through a 0.22 μm pore membrane filter. Subsequently, 1 mL of each extraction, diluted up to 3 mL with distilled water, was analyzed by HPAEC (High-Performance Anion Exchange Chromatography) with a carbopac PA-1 ion exchange column (4.0 mm × 250 mm; Thermo Scientific Dionex, Sunnyvale, CA, United States) and a Dionex ICS-3000 system (Thermo Scientific Dionex, Sunnyvale, CA, United States).

#### Determination of Molecular Weight

High-performance gel permeation chromatography (1100 Series HPLC, Agilent) was used to analyze the molecular weights of the polysaccharide fractions. The HPLC was equipped with a differential refraction index detector and a TSK-G3000 PW_*XL*_ column constantly kept at 30°C. Dextran standards of different molecular weights (Dextran T1000, T500, T70, T40, T10, and T5) were examined in turn to plot the calibration curve according to the retention time and the logarithm of the corresponding molecular weights.

#### Fourier-Transform Infrared Analysis

Fourier transform infrared (FT-IR) spectra of every dried sample were determined using the KBr-pellet method with a Nicolet iN10 FT-IR spectroscope (Thermo Nicolet Corporation, Madison, WI, United States), at frequencies ranging from 4,000 cm^–1^ to 500^–1^ with a resolution of 4 cm^–1^ (17).

#### Methylation Analysis

The uronic acid reduction was carried out according to the literature with minor modifications (18). 20 mg of polysaccharide SSP-2 was dissolved in 5 mL of ice-cold 1 M imidazole-HCl, and 1 mL of 100 mg/mL NaBD_4_ was added three times. Methylation with methyl iodide (CH_3_I) was performed according to the published report (19). The carboxyl-reduced sample was then dissolved in 50 μm of DMSO/NaOH (60°C) slurry 50% (wt/wt) without ultrasonic treatment. Once ionized, the polysaccharide was treated with CH_3_I. The methylated residue was hydrolyzed by treatment with 2 mL of 2 M TFA at 100°C for 6 h and was evaporated to get rid of the excess acid. The acquisition was finally acetylated with acetic anhydride and pyridine at 100°C for 1 h with the addition of NaBH_4_ as a reducing agent. After acetylation, the resulting alditol acetates were analyzed with GC-MS (gas chromatography-mass spectrometry) system equipped with a DB-5 capillary column (0.25 mm × 30 m × 0.25 m, Thermo Finnigan Co., Santa Clara, CA, United States).

#### 1D and 2D NMR Spectroscopy

1D nuclear magnetic resonance (NMR) spectra analysis of SSP-2 was carried out using a 500 MHz Bruker Avance III nuclear magnetic resonance instrument (Germany) with each sample dissolved in 1 mL of 99.8% D_2_O. Two-dimensional spectra, including HSQC (heteronuclear single quantum coherence spectroscopy), COSY (^1^H-^1^H correlated spectroscopy), and HMBC (^1^H detected heteronuclear multiple bond correlation spectroscopy) experiments were also ascertained by standard Bruker procedures at 25°C according to our previous method (20).

#### Thermal Analysis

Thermogravimetric analyses were performed on an SDT Q600 Simultaneous Thermal Analyzer (TA Instruments, United States) in a nitrogen atmosphere at 100 mL/min and scanned from 45°C to 700°C at a heating rate of 10°C/min.

#### DPPH Radicals Scavenging Activity

The 2,2 Diphenyl-1-picrylhydrazyl (DPPH) radicals scavenging activity of SSPs was measured according to the previous report (21). Briefly, 1.0 mL of SSPs solution at different concentrations was added to 3.0 mL 0.1 mM DPPH in methanol and incubated at room temperature for 60 min in the dark. The absorbance was determined using a microplate reader at 517 nm, and ascorbic acid was used as a positive control and antioxidant standard. The DPPH radical scavenging activity was calculated according to the following equation:


$$\mathrm{DPPH}\mathrm{radical}\mathrm{scavenging}\mathrm{activity}\left(\%\right)=\left[1-\left(A_{2}-A_{1}\right)/A_{0}\right]100\%$$

Where A_0_ is the absorbance of the blank, in which distilled water was used in place of the sample, A_1_ is the absorbance of the test sample solution, but the dehydrated alcohol instead of the DPPH solution. A_2_ is the absorbance of the sample solution.

#### Iron Chelating Activity

The iron chelating activity of the polysaccharides was evaluated according to the method described by Shao et al. (22). Briefly, the reaction mixture containing 50 μL of SSPs at various concentrations, 100 μL of FeSO_4_⋅7H_2_O (0.5 mM), and 50 μL of ferrozine (5.0 mM), was distributed into a microplate. The mixture was incubated at room temperature for 30 min, using EDTA-2Na as the blank. After that, the absorbance was determined immediately at 562 nm. The iron chelation potential was expressed as the percentage inhibition and was calculated by the following equation:


$${\mathrm{Fe}}^{2+}\mathrm{chelating}\mathrm{ability}\left(\%\right)=\left(1-A_{S}/A_{0}\right)100\%$$

Where A_*S*_ is the absorbance of the sample, and A_0_ is the absorbance of the blank, in which distilled water was used in place of the sample.

## Results and Discussion

### Reclamation and Purification of Polysaccharide Fractions

**FIGURE 1:**
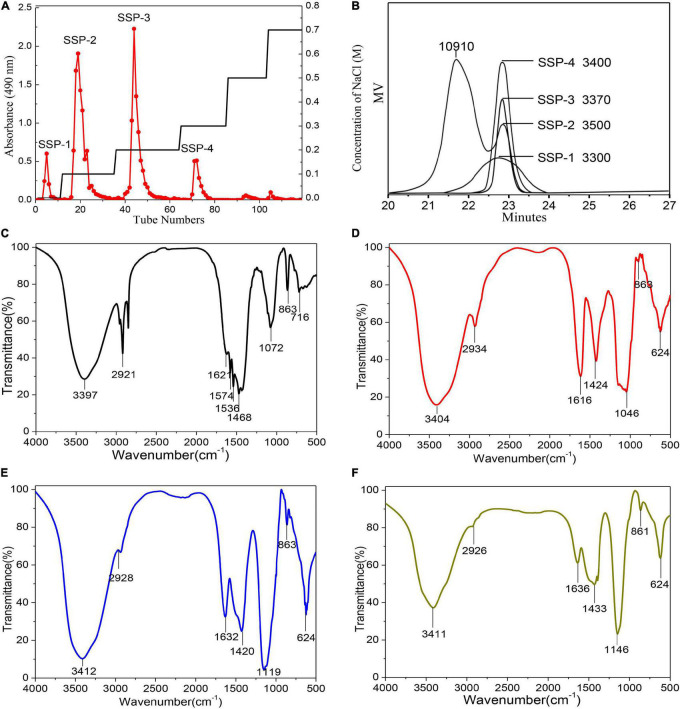
Elution profile and physicochemical analysis: DEAE cellulose-52 column elution profile **(A)**; GPC profiles and molecular weights **(B)**; FT-IR spectra of SSP-1 **(C)**, SSP-2 **(D)**, SSP-3 **(E)**, and SSP-4 **(F)**.

The hot-alkaline waste liquor was obtained simulating the soaking process of wet decortication as described in section “Sesame Wet Decortication Waste Liquor Collection.” The total yield of the crude polysaccharide SSP from the waste liquor was about 0.3% (based on dry matter of the sesame seeds) after extraction, ethanol precipitation, removal of proteins, dialysis, and lyophilization. Four main fractions were produced by fractionation with distilled water and NaCl solutions and were collected by preparative size-exclusion chromatography on a DEAE-52 column based on the total carbohydrate elution profile (Figure 1A). Following concentration, dialysis, and lyophilization, SSP-1, SSP-2, SSP-3, and SSP-4 fractions were finally obtained. They accounted for 15.7, 53.9, 12.1, and 11.8% of the total SSP content, respectively. SSP-1, SSP-3, and SSP-4 were each eluted as single and sharp symmetrical peaks, which indicated their homogeneity. In contrast, the main fraction SSP-2 showed a shoulder on both the DEAE-52 column and GPC chromatographs, suggesting it was a polydispersed system composed of more than one compound.

### Monosaccharide Compositions of the Polysaccharides

For the monosaccharide composition analysis, the molar content of each monosaccharide preparation was identified by comparison with monosaccharide standards; results are given in Table 1. The result indicated that all of the sesame polysaccharide fractions were rich in galactose and xylose, with notable differences in other chemical components. For instance, the monosaccharides of SSP-1, which was eluted by distilled water, markedly differed from the other three fractions. SSP-1 was neutral with no acidic sugar detected. The other fractions (SSP-2, SSP-3, and SSP-4) eluted at concentrations of 0.1, 0.2, and 0.3 M NaCl, respectively, and contained 8.2, 4.4, and 7.5% uronic acid, respectively. These results match those obtained by Hokputsa (23). Furthermore, glucose (28.4%) and mannose (11.2%) were the dominant components in SSP-1, while arabinose (29.1%) and galactose (23.9%) were the dominant components in SSP-2. Both SSP-3 and SSP-4 were mainly made up of galactose and xylose in molar ratios of 33.4:42.6 and 37.3:21.5, respectively. The high percentages of galactose, xylose, and arabinose with some mannose in the SSP fractions were considered to indicate correspondingly more arabinoxylan and galactomannan in sesame polysaccharides, whereas other sugars, presenting in lower amounts, might originate from different kinds of polysaccharides (24).

**TABLE 1:** Composition of monosaccharides in polysaccharides obtained from the waste liquor produced by wet decortication of sesame seeds.

Fractions	Monosaccharide composition (%)							
	Rha^a^	Ara^a^	Gal^a^	Glc^a^	Xyl^a^	Man^a^	GlcA^a^	GalA^a^
SSP-1	9.3	5.9	29.8	28.4	15.4	11.2	nd^b^	nd^b^
SSP-2	16.3	29.1	23.9	9.0	13.5	nd^b^	6.2	2.0
SSP-3	nd^b^	14.6	33.4	5.0	42.6	nd^b^	4.4	nd^b^
SSP-4	15.1	8.7	37.3	5.6	21.5	4.2	4.5	3.0

*^a^Rha, rhamnose; Ara, arabinose; Gal, galactose; Glc, glucose; Xyl, xylose; Man, mannose; GlcA, glucuronic acid; GalA, galacturonic acid.*

*^b^nd, not detected.*

### Average Molecular Weight

The homogeneity and average molecular mass of each purified fraction were calculated based on the calibration curve made with a group of standard dextran determined by HPLC. As shown in Figure 1B, molar mass distributions of SSP-1, SSP-3, and SSP-4 as shown by HPLC had narrow and symmetrical peaks, thus confirming their homogeneity. According to the calibration curve, l g Mw = -0.33t + 10.9 (*R*^2^ = 0.996) (where t is the retention time, and *R*^2^ represents the fitting coefficient); the average molecular weights of SSP-1, SSP-3, and SSP-4 were determined to be 3,300, 3,370, and 3,400 Da, respectively. However, the HPLC profile of SSP-2 appeared to be bimodal in shape. SSP-2 consisted of a polysaccharide calculated as 10,910 Da with a retention time of 21.2 min and another calculated as 3,500 Da with a retention time of 22.7 min. SSP-2 represented a large proportion of SSP, owing to both its high molecular weight and high content, shown as the large peak area in the elution profile. Nastaran et al. (25) suggested that the presence of NaOH would increase the degradation of the polysaccharides, thereby also increasing the quantity of low molecular weight (<50 kDa) polysaccharides. Carbonell-Barrachina et al. (8) observed that alkaline extraction relatively easily dissolved the polysaccharides with high molecular weights, while the lower-molecular-weight polysaccharide fractions were likely to be extracted with milder conditions like DMSO treatment (26). Thus, one possible explanation for the relatively low molecular weight is that the mild dehulling conditions combined with the alkaline degradation of polysaccharides counteracted the extraction effects of alkali liquor.

### Fourier-Transform Infrared Analysis

Fourier-Transform Infrared spectroscopy, an effective analytical technique, is based on the fact that bonds and functional groups vibrate at characteristic frequencies (27). As shown in Figures 1C–F, the purified fractions from SSP had similar characteristic absorption peaks at around 3,397, 2,921, 1,632, 1,420, 1,119, 863, and 624 cm^–1^, indicating that the structure did not obviously change during elution of the polysaccharides. The FT-IR spectra showed a strong and broadband at 3,411 cm^–1^ assigned to the O–H stretching vibration of the polysaccharides, while the peak at 2,926 cm^–1^ was attributed to the C–H stretching vibration (28). In good accord with the sugar composition, in the spectra for all the fractions except SSP-1, the symmetrical and asymmetrical stretching vibrations of the carboxylate group similarly showed two clear intense bands at 1,424 and 1,632 cm^–1^ (29, 30). Furthermore, the carbohydrates showed high absorbencies within the region of 1,200–1,000 cm^–1^, indicating the presence of galactopyranose in the main chain (31, 32). The spectra of all four polysaccharides lacked a signal at 1,730 cm^–1^ for carbonyl stretching, which indicated that the mild dehulling process entirely saponified the acetyl groups and methyl esters.

### Methylation Analysis

As listed in Table 1, SSP-2 was composed of rhamnose, arabinose, galactose, glucose, xylose, glucuronic acid, and galacturonic acid in the molar ratio of 16.3:29.1:23.9:9.0:13.5:6.2:2.0. To determine the monosaccharide linkages, SSP-2 was further methylated and hydrolyzed into alditol acetate derivatives, and then determined by methylation analysis (shown in Table 2 and Supplementary Figure 1). The analysis indicated that the dominant modes of linkages were →5)-_L_-Ara*f*-(1→, →2,4)-_D_-Xyl-*p*-(1→, →2)-_D_-Glc*p*A-(1→, →4)-_D_-Glc*p*-(1→ and →6)-_D_-Gal*p*-(1→, accompanied by small amounts of →2,3,4)-_D_-Xyl*p*-(1→, →2)-_L_-Rha*p*-(1→, →4)-_L_-Ara*p*-(1→, →3,5)-_L_-Ara*f*-(1→, →3)-_D_-Gal*p*-(1→, →3,6)-_D_-Glc*p*-(1→ and →2,4)-_D_-Gal*p*-(1→. The major terminal linkages, which attached to the branch-points of the backbone, were detected as T-_D_-Gal*p*, T-_L_-Ara*f* and T-_L_-Rha*p* residues. A tiny bit of →3)-_L_-Rha*p*-(1→, →3,4)-_L_-Rha*p*-(1→ and →2,3)-_L_-Ara*p*-(1→ were also detected (each contain <2.2% of the total peak area). The results showed that the repeating elements of SSP-2 mainly consisted of a (1→5)-Ara*f* backbone with branches consisting of many (1→2,4)-Xyl*p* residues attached to O-3 of some xylose or a (1→2,4)-Xyl*p* backbone with branches consisting of many (1→5)-Ara*f* residues attached to O-4 of some xylose residues. The existence of large quantities of (1→2,3,4)-linked xylose suggested that the SSP-2 fraction is highly branched. According to the relative amounts of (1→2,3,4)-linked xylose, the theoretical branch ratios at the C-3 of the backbone arabinose may be approximately 64.8%. Similarly, 38.7% of (1→5)-linked arabinose were substituted at O-3 positions to form (1→3,5)-linked arabinose moieties. Theoretically, 32.0% of the glycosyl residues presented as branch points, and 30.9% of terminal residues were detected. The result was in accord with the expectation that the sum of all the terminal residues should be roughly equal to the sum of branch point residues (33). According to the result, although not entirely identical, the proportions of the methylated derivatives were consistent with the polysaccharide monosaccharide composition.

**TABLE 2:** Methylation analysis of SSP-2.

Methylated sugar	Linkages	Molar ratio (%)
2,3,4-Me_3_-Rha*p*^a^	T-_L_-Rha*p*-(1→	8.9
3,4-Me_2_-Rha*p*	→2)-_L_-Rha*p*-(1→	4.6
2,4-Me_2_-Rha*p*	→3)-_L_-Rha*p*-(1→	2.0
2-Me_1_-Rha*p*	→3,4)-_L_-Rha*p*-(1→	2.2
2,3,5-Me_3_-Ara*f*	T-_L_-Ara*f*-(1→	9.0
2,3-Me_2_-Ara*p*	→4)-_L_-Ara*p*-(1→	4.3
2,3-Me_2_-Ara*f*	→5)-_L_-Ara*f*-(1→	10.4
4-Me_1_-Ara*p*	→2,3)-_L_-Ara*p*-(1→	1.8
2-Me_1_-Ara*f*	→3,5)-_L_-Ara*f*-(1→	4.0
2,3,4,6-Me_4_-Gal*p*	T-_D_-Gal*p*-(1→	13.0
2,4,6-Me_3_-Gal*p*	→3)-_D_-Gal*p*-(1→	3.8
2,3,4-Me_3_-Gal*p*	→6)-_D_-Gal*p*-(1→	5.4
3,6-Me_2_-Gal*p*	→2,4)-_D_-Gal*p*-(1→	2.6
2,3,6-Me_3_-Glc*p*	→4)-_D_-Glc*p*-(1→	5.7
2,4-Me_2_-Glc*p*	→3,6)-_D_-Glc*p*-(1→	3.3
3-Me_1_-Xyl*p*	→2,4)-_D_-Xyl*p*-(1→	7.7
Xyl*p*^b^	→2,3,4)-_D_-Xyl*p*-(1→	5.0
3,4,6-Me_3_-Glc*p*A	→2)-_D_-Glc*p*A-(1→	6.2

*^a^2,3,4-Me_3_-Rhap, 1,5-di-O-acetyl-6-deoxy-2,3,4-tri-O-methyl-_L_-mannitol, ect.*

*^b^Xylp = 1,2,3,4,5-penta-O-acetyl xylitol.*

### NMR Spectroscopy

**FIGURE 2:**
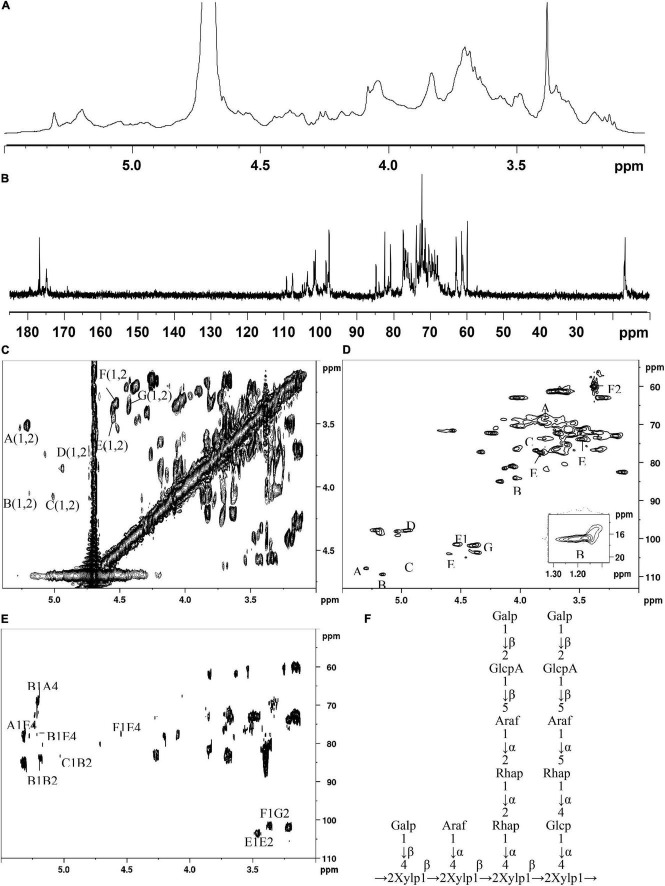
^1^H **(A)**, ^13^C **(B)**, COSY **(C)**, HSQC **(D)**, and HMBC **(E)** spectra of SSP-2 in D_2_O; proposed structure of SSP-2 **(F)**.

The main structural features of SSP-2 were further confirmed from the results of a combination of 1D and 2D NMR analysis, shown in Figures 2A–E, which revealed detailed structural information such as α- or β- configurations, monosaccharide composition, and sugar linkage patterns (34). The ^1^H NMR spectrum (Figure 2A) demonstrated that the chemical shifts of anomeric protons present in the region from 4.43 to 5.30 ppm were higher or lower than 5.0 ppm, which indicated that both α- and β- anomeric configurations were present in the SSP-2 fraction (35). The typical signals at the high field (1.17–1.23 ppm) were attributed to the –CH_3_ (C6) of rhamnose (36). The ^13^C NMR spectrum (Figure 2B) of SSP-2 showed anomeric carbon signals corresponding to the proton signals from 97.68 to 109.34 ppm and multiple non-anomeric carbon signals in the region of 59.90 to 84.87 ppm. The corresponding chemical shifts of rhamnose for –CH_3_ (C6) at 16.77 and 16.91 ppm in the ^13^C NMR spectrum can be assigned according to the HSQC spectrum (Figure 2D). Furthermore, the peaks in the range of 174.99 to 176.87 ppm could be attributed to the carboxyl carbons of Glc*p*A (37).

The detailed sugar ring carbon/hydrogen signals were determined as far as possible by combining the results of methylation analysis, HSQC, COSY, and HMBC experiments with the literature data. In addition, the relative configuration of the residues was established on the basis of the characteristic chemical shifts of the anomeric carbons. By comparison with the results of the HSQC experiment, seven clear signals in the anomeric region of the ^1^H NMR spectrum were observed, which could be attributed to anomeric protons. These seven resonances at 4.43–5.30 ppm were designated as A (H1)-G (H1) according to the decreasing chemical shifts of their anomeric protons. Other chemical shifts of sugar residues were not detected in the spectrum as described in the methylation analysis due to their relatively low content. To further confirm the detailed proton signals (Table 2), the corresponding sugar residues’ chemical shifts were assigned based on the COSY spectrum (Figure 2C) and the literature. The proton chemical shifts of residues A, B, F, and G showed H-1 up to H-6 correlations, and the other residues (C, D, and E) were assigned in turn from H-1 to H-5 (Table 3). In succession, the carbon signals corresponding to the assigned protons were identified from the cross-peaks in the HSQC spectrum. The corresponding residues were tentatively assigned to (1→4)-α-_D_-Glc*p* (A), (1→2)-α-_L_-Rha*p* (B), (1→5)-α-_L_-Ara*f* (C), T-α-_L_-Ara*f* (D), (1→2,4)-β-_D_-Xyl*p* (E), T-β-_D_-Gal*p* (F) and (1→2)-β-_D_-Glc*p*A (G), which was consistent with the methylation results and literature data ((38); Q. (37, 39–42)). According to the linked positions of the residues shown by the cross-peaks, the chemical shifts of individual residues produced by ^1^H-^1^H COSY and HSQC spectra were verified by the HMBC experiments. For instance, the H-5/C-6 signal of a long-range correlation appeared at 4.23/176.87 ppm in the HMBC spectrum (Figure 2E); hence the corresponding residue of this sugar could be identified as Glc*p*A.

**TABLE 3:** Summary of ^1^H and ^13^C chemical shifts (δ,ppm) for SSP-2.

Residues	H1/C1	H2/C2	H3/C3	H4/C4	H5/C5	H6/C6
A: →4)-α-_D_-Glc*p*-(1→	5.30	3.53	3.35	3.83	3.95	3.25
	107.76	77.17	71.40	68.79	69.57	72.31
B: →2)-α-_L_-Rha*p*-(1→	5.20	4.06	3.82	3.35	3.78	1.23
	109.34	83.95	73.71	62.86	70.05	16.77
C: →5)-α-_L_-Ara*f*-(1→	5.02	4.08	3.71	4.21	3.83	
	107.65	80.89	68.80	70.43	76.71	
D: T-α-_L_-Ara*f*-(1→	4.94	3.86	4.19	3.64	3.84	
	97.80	78.84	68.50	69.82	81.60	
E:→2,4)-β-_D_-Xyl*p*-(1→	4.56	3.45	3.64	3.86	4.08	
	103.85	73.85	69.66	77.27	80.89	
F: T-β-_D_-Gal*p*-(1→	4.53	3.35	4.07	3.87	4.19	3.65
	101.46	72.12	76.23	73.64	81.40	72.57
G: →2)-β-_D_-Glc*p*A-(1→	4.43	3.37	3.57	3.73	3.15	4.23
	101.91	59.87	71.40	70.05	82.41	176.87

Quite apart from confirming the above assignments, HMBC NMR analysis was used to clarify the linkages between the structural fragments. Both intra- and inter-connections among these different sugar residues were determined according to all the information given by the cross-peaks between anomeric protons and non-anomeric carbons of every sugar residue in HMBC. The cross peak for H-1 of (1→4)-α-_D_-Glc*p* and C-4 of (1→2,4)-β-_D_-Xyl*p* was observed, indicating that (1→4)-α-_D_-Glc*p* is connected to the O-4 position of (1→2,4)-β-_D_-Xyl*p*. Similarly, H-1 of (1→2)-α-_L_-Rha*p* is linked to the O-4 position of (1→4)-α-_D_-Glc*p* and (1→2,4)-β-_D_-Xyl*p*; both H-1 of (1→2)-α-_L_-Rha*p* and (1→5)-α-_L_-Ara*f* are connected to the O-2 position of (1→2)-α-_L_-Rha*p*; and H-1 of (1→2,4)-β-_D_-Xyl*p* is linked to its O-2 position. H-1 of T-α-_L_-Ara*f* was linked to the O-4 position of (1→2,4)-α-_D_-Xyl*p* as indicated by a cross peak at 4.94/77.27 ppm (H-1 of Ara*f* and C-4 of (1→2,4)-α-_D_-Xyl*p*), and (1→2)-β-_D_-Glc*p*A were linked to the O-5 position of (1→5)-α-_L_-Ara*f* at 4.43/76.71 ppm (H-1 of Glc*p*A and C-5 of Ara*f*). Similarly, H-1 of T-β-_D_-Gal*p* was both connected to the O-4 position of (1→2,4)-α-_D_-Xyl*p* and the O-2 position of (1→2)-β-_D_-Glc*p*A as indicated by a cross peak at 4.53/77.27 ppm (H-1 of Gal*p* and C-5 of (1→2)-β-_D_-Glc*p*A) and another at 3.37/101.46 ppm (H-1 of Gal*p* and C-2 of (1→2)-β-_D_-Glc*p*A). And the cross peak present at 1.23/70.05 ppm (H-6 of and C-5 of Rha*p*) concurred with the structure of (1→2)-α-_L_-Rha*p*. Based on the results above, a possible structure for SSP-2 is proposed as shown in Figure 2F. It is difficult to distinguish all the linkage patterns of the polysaccharide *via* NMR owing to its high degree of branching.

### Thermal Analysis

Thermal stability is a critical characteristic that determines applications. TG and DTG analyses were conducted to assess the thermal behavior of the samples and to figure out how thermal transitions associated with evaporation, decomposition, and oxidation could affect the structure (43). The residual mass of the prepared samples was measured as a function of temperature, as shown in Supplementary Figure 2. Peak temperatures for all of the derivative curves similarly showed two main stages of loss of mass. These values were susceptible to changes depending on the chemical composition, degrees of polymerization, molecular weight, and branching of the samples. There was a weight loss less than 17% at the first stage in the period of 100–150°C, which could be explained by the dissociation of absorbed and structural water and which coincided with the existence of hydrophilic groups in each polysaccharide (44). The second stage, including endothermic peaks between 150 and 550°C, was due to the heat degradation of the polysaccharides. Differences among samples could be observed in this stage. The results showed that SSP-1, with a higher Man/Gal ratio, had a high decomposition temperature, which was consistent with the literature (45). Na and Lee showed that high mannose content resulted in the greater binding energy of the polysaccharide chains. The slower degradation at the end of the second stage finished at 550°C with a large quantity of char, which was related to the inorganic compounds and volatile decomposition products in the samples, such as hydroxyl and carboxyl groups (46).

### Antioxidant Activity and the Structure-Activity Relationship

#### DPPH Scavenging Activity

**FIGURE 3:**
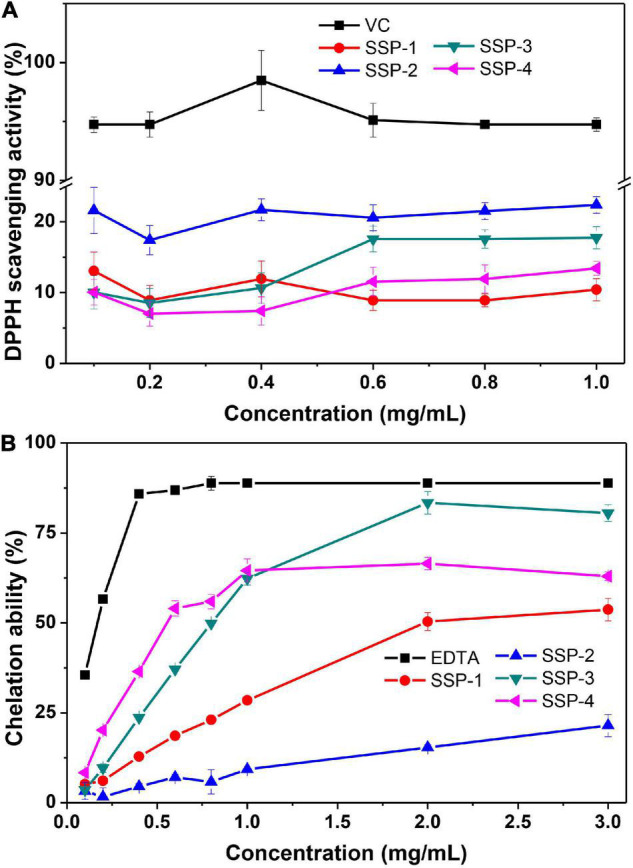
DPPH radical scavenging assay **(A)** and ferrous ion chelating ability assay **(B)**.

The scavenging of DPPH radicals could be used to indicate the hydrogen-donating ability of the purified polysaccharides. As shown in Figure 3A, the scavenging activity of SSP-1, SSP-2, SSP-3, SSP-4, and V_*C*_ against the DPPH radical was monitored at 517 nm. As can be seen, SSP-2 showed better scavenging activity of DPPH radicals than the other three polysaccharides. However, the scavenging activity in all concentrations ranging from 0.1 to 1.0 mg/mL was no more than 25%, which was much lower than that of Vc. Niu (47) reported that the alkali-soluble heteropolysaccharide from *pentaphyllum* Makino showed lower absorbance capacities for DPPH than the water-soluble polysaccharide and the DPPH radical scavenging capacity of the alkali-soluble polysaccharide was calculated to be 2 μmol TE/g. The chemical structures, molecular weights, and monosaccharide compositions of the alkali-soluble polysaccharide were different from the polysaccharide extracted with hot water, which will result in different antioxidant activities.

#### Iron Chelating Activity

Fe^2+^ is a powerful pro-oxidant with active reactivity and it involves in the Fenton reaction which leads to the oxidation of lipid and protein. Therefore, the chelating activity of polysaccharides can be used as an obvious indicator of antioxidant capacity (48). The correlation between the concentration of the polysaccharides and their chelating activities to Fe^2+^ is shown in Figure 3B. This result indicated that the SSPs represent concentration-dependent chelation ability and SSP-3 had the best chelating ability with a chelating rate of 80.5% at the concentration of 3.0 mg/mL. At concentration of 3.0 mg/mL, the chelating rates of SSP-1, SSP-2 and SSP-4 were 53.7.5, 21.5, and 63.0%, respectively. The results indicated that all of the SSPs possessed powerful Fe^2+^ chelating ability, which is still weaker than that of EDTA. The formation of SSPs -Fe^3+^ complex inhibited the occurrence of Fenton reaction, therefore the generation of hydroxyl radical is reduced. However, the way in which structure affects the antioxidant activities remains unknown.

#### Correlation of Structure and Antioxidant Activity

The biological activities of polysaccharides were mainly related to their molecular weight, monosaccharide composition, sulfate content, glycosidic linkage, and chain structure (49). Although the relationships between function and structure of polysaccharides remained unclear, some evidence can be inferred from our study as follows.

It is widely known that polysaccharides possess antioxidant effects due to the number of reductive hydroxyl groups in their structure. And polysaccharides with low molecule weight is more likely to present a stronger reducing power, because they may have a higher content of reducing end (available active hydroxyl group terminals) to accept and eliminate the free radicals. However, molecule weight is certainly not the deciding factor of the antioxidant effects. In our study, the SSP-2 fraction with a higher molecule weight (10.9 kDa) presented better DPPH scavenging activity than the other fractions, which was consistent with an investigation by Liang et al. (50). It was found that fraction MDP-2 with a higher molecule weight (1,700 kDa) from the *Mycena dendrobii* has better antioxidant activity than fraction MDP-1 (602 kDa).

In addition, the discrepancies of biological activities between the four fractions were probably owing to the difference in the ratio of monosaccharides. Their DPPH scavenging activities are in the order of ascorbic acid > SSP-2 > SSP-3 > SSP-4 > SSP-1, suggesting the probable relationship between the polysaccharides and their xylose content. And SSP-2 performed better in DPPH scavenging activities may be due to its high uronic acid content (51). At concentration of 3.0 mg/mL, the ferrous ion chelating activity of SSP-1, SSP-2, SSP-3, and SSP-4 are concentration-dependent and are in the order of SSP-3 > SSP-4 > SSP-1 > SSP-2. In addition, the xylose content in them was determined in the same order, indicating that the chelating ability of the four polysaccharides was probably due to their xylose content.

## Conclusion

Four polysaccharide fractions (SSP-1, SSP-2, SSP-3, and SSP-4) were purified from the waste liquor obtained by simulating the soaking process of wet decortication of sesame seeds. Structural analysis revealed that the polysaccharides reclaimed from this process were low-molecular-weight polysaccharides. The main fraction, SSP-2, consisted of rhamnose, arabinose, galactose, glucose, xylose, glucuronic acid, and galacturonic acid in the molar ratio of 16.3:29.1:23.9:9.0:13.5:6.2:2.0. The results of monosaccharide composition, methylation, and NMR spectroscopic analyses indicated that the backbone of SSP-2 mainly consisted of (1→2,4)-β-_D_-Xyl*p* residues with side chains connected to the O-4 position composed of a great deal of T-β-_D_-Gal*p* and (1→5)-α-_L_-Ara*f* residues, and lesser contents of (1→4)-α-_D_-Glc*p*, (1→2)-α-_L_-Rha*p*, T-α-_L_-Ara*f*, and (1→2)-β-_D_-Glc*p*A residues. Polysaccharides from the waste liquor have complex structures and may have potential as an ingredient in foods or as excipients in pharmaceuticals, and these possibilities merit further study. In addition, removing polysaccharides from the waste liquor will simplify wastewater treatment. Further studies on the bioactivity and chemistries of these four polysaccharide fractions are in progress.

## Data Availability Statement

The raw data supporting the conclusions of this article will be made available by the authors, without undue reservation.

## Author Contributions

Y-RL: conceptualization, writing-original draft preparation, and methodology. SX: data curation and visualization. R-YZ and M-XY: investigation. H-ML: writing-review and editing and validation. X-DW: supervision and writing-review and editing. All authors contributed to the article and approved the submitted version.

## Conflict of Interest

The authors declare that the research was conducted in the absence of any commercial or financial relationships that could be construed as a potential conflict of interest.

## Publisher’s Note

All claims expressed in this article are solely those of the authors and do not necessarily represent those of their affiliated organizations, or those of the publisher, the editors and the reviewers. Any product that may be evaluated in this article, or claim that may be made by its manufacturer, is not guaranteed or endorsed by the publisher.

## References

[1] Mikropoulou EV Petrakis EA Argyropoulou A Mitakou S Halabalaki M Skaltsounis LA. Quantification of bioactive lignans in sesame seeds using HPTLC densitometry: comparative evaluation by HPLC-PDA. *Food Chem.* (2019) 288:1–7. 10.1016/j.foodchem.2019.02.109 30902268

[2] Liu HM He MK Yao YG Qin Z Cai XS Wang XD. Pectic polysaccharides extracted from sesame seed hull: physicochemical and functional properties. *Int J Biol Macromol.* (2021) 192:1075–83. 10.1016/j.ijbiomac.2021.10.077 34673100

[3] Mohamed IA Uslu N Musa Ozcan M Al Juhaimi F Ghafoor K Babiker EE Effect of conventional oven roasting treatment on the physicochemical quality attributes of sesame seeds obtained from different locations. *Food Chem.* (2021) 338:128109. 10.1016/j.foodchem.2020.128109 33091991

[4] Rangkadilok N Pholphana N Mahidol C Wongyai W Saengsooksree K Nookabkaew S Variation of sesamin, sesamolin and tocopherols in sesame (*Sesamum indicum* L.) seeds and oil products in Thailand. *Food Chem.* (2010) 122:724–30. 10.1016/j.foodchem.2010.03.044

[5] Besharati M Palangi V Taghizadeh A Kaya A Abachi S. Determining the effect of natural inhibitors on sesame meal degradability using in vitro three step method. *Vet Arh.* (2021) 91:513–21. 10.24099/vet.arhiv.1138

[6] Eom SJ Zu HD Lee J Kang MC Park J Song KM Development of an ultrasonic system for industrial extraction of unheated sesame oil cake. *Food Chem.* (2021) 354:129582. 10.1016/j.foodchem.2021.129582 33756313

[7] Moharram YG Osman HOA Abou-Ei-Khier YIA. Wet decortication of sesame seeds by new methods. *Food Nutr Bull.* (1990) 12:1–6. 10.1177/156482659001200126

[8] Carbonell-Barrachina AA Lluch MA Perez-Munera I Hernando I Castillo S. Effects of chemical dehulling of sesame on color and microstructure. *Food Sci Technol Int.* (2009) 0:1–6. 10.1177/1082013209339704

[9] Elleuch M Besbes S Roiseux O Blecker C Attia H. Quality characteristics of sesame seeds and by-products. *Food Chem.* (2007) 103:641–50. 10.1016/j.foodchem.2006.09.008

[10] Yu Y Shen M Song Q Xie J. Biological activities and pharmaceutical applications of polysaccharide from natural resources: a review. *Carbohydr Polym.* (2018) 183:91–101. 10.1016/j.carbpol.2017.12.009 29352896

[11] Guo R Chen M Ding Y Yang P Wang M Zhang H Polysaccharides as potential anti-tumor biomacromolecules –a review. *Front Nutr.* (2022) 9:838179. 10.3389/fnut.2022.838179 35295918 PMC8919066

[12] Kalita P Ahmed AB Sen S Chakraborty R. A comprehensive review on polysaccharides with hypolipidemic activity: occurrence, chemistry and molecular mechanism. *Int J Biol Macromol.* (2022) 206:681–98. 10.1016/j.ijbiomac.2022.02.189 35247430

[13] Zhou S Huang G. Extraction, derivatization, and antioxidant activity of *Morinda citrifolia* polysaccharide. *Chem Biol Drug Des.* (2022) 99:603–8. 10.1111/cbdd.14023 35092172

[14] Ji X Guo J Ding D Gao J Hao L Guo X Structural characterization and antioxidant activity of a novel high-molecular-weight polysaccharide from *Ziziphus jujuba* cv. Muzao. *J Food Meas Charact.* (2022) 16:2191–200. 10.1007/s11694-022-01288-3

[15] Huo S Wang H Chen J Hu X Zan X Zhang C A preliminary study on polysaccharide extraction, purification, and antioxidant properties of sugar-rich filamentous microalgae *Tribonema minus*. *J Appl Phycol.* (2022) 48:1–13. 10.1007/s10811-021-02630-w

[16] Dubois M Gilles KA Hamilton JK Rebers PA Smith F. Colorimetric method for determination of sugars and related substances. *Anal Chem.* (1956) 28:350–6. 10.1021/ac60111a017

[17] Ji X Guo J Pan F Kuang F Chen H Guo X Structural elucidation and antioxidant activities of a neutral polysaccharide from arecanut (*Areca catechu* L.). *Front Nutr.* (2022) 9:853115. 10.3389/fnut.2022.853115 35340550 PMC8948432

[18] Kim JB Carpita NC. Changes in esterification of the uronic acid groups of cell wall polysaccharides during elongation of maize coleoptiles. *Plant Physiol.* (1992) 98:646–53. 10.1104/pp.98.2.646 16668690 PMC1080239

[19] Pettolino FA Walsh C Fincher GB Bacic A. Determining the polysaccharide composition of plant cell walls. *Nat Protoc.* (2012) 7:1590–607. 10.1038/nprot.2012.081 22864200

[20] Wang L Liu HM Xie AJ Wang XD Zhu CY Qin GY. Chinese quince (*Chaenomeles sinensis*) seed gum: structural characterization. *Food Hydrocolloids.* (2018) 75:237–45. 10.1016/j.foodhyd.2017.08.001

[21] Chen J Zhang X Huo D Cao C Li Y Liang Y Preliminary characterization, antioxidant and alpha-glucosidase inhibitory activities of polysaccharides from *Mallotus furetianus*. *Carbohydr Polym.* (2019) 215:307–15. 10.1016/j.carbpol.2019.03.099 30981359

[22] Shao LL Xu J Shi MJ Wang XL Li YT Kong LM. Preparation, antioxidant and antimicrobial evaluation of hydroxamated degraded polysaccharides from *Enteromorpha prolifera*. *Food Chem.* (2017) 237:481–7. 10.1016/j.foodchem.2017.05.119 28764023

[23] Hokputsa S Harding SE Inngjerdingen K Jumel K Michaelsen TE Heinze T Bioactive polysaccharides from the stems of the Thai medicinal plant *Acanthus ebracteatus*: their chemical and physical features. *Carbohydr Res.* (2004) 339:753–62. 10.1016/j.carres.2003.11.022 14980816

[24] Coll-Almela L Saura-Lopez D Laencina-Sanchez J Schols HA Voragen AG Ros-Garcia JM. Characterisation of cell-wall polysaccharides from mandarin segment membranes. *Food Chem.* (2015) 175:36–42. 10.1016/j.foodchem.2014.11.118 25577048

[25] Khodaei N Karboune S. Extraction and structural characterisation of rhamnogalacturonan I-type pectic polysaccharides from potato cell wall. *Food Chem.* (2013) 139:617–23. 10.1016/j.foodchem.2013.01.110 23561153

[26] Bian J Peng F Xu F Sun RC Kennedy JF. Fractional isolation and structural characterization of hemicelluloses from *Caragana korshinskii*. *Carbohydr Polym.* (2010) 80:753–60. 10.1016/j.carbpol.2009.12.023

[27] Long X Hu X Xiang H Chen S Li L Qi B Structural characterization and hypolipidemic activity of *Gracilaria lemaneiformis* polysaccharide and its degradation products. *Food Chem. X.* (2022) 14:100314. 10.1016/j.fochx.2022.100314 35492254 PMC9046617

[28] Ji X Liu F Peng Q Wang M. Purification, structural characterization, and hypolipidemic effects of a neutral polysaccharide from *Ziziphus jujuba* cv. Muzao. *Food Chem.* (2018) 245:1124–30. 10.1016/j.foodchem.2017.11.058 29287331

[29] Saravana PS Cho YN Patil MP Cho YJ Kim GD Park YB. Hydrothermal degradation of seaweed polysaccharide: characterization and biological activities. *Food Chem.* (2018) 268:179–87. 10.1016/j.foodchem.2018.06.077 30064746

[30] Wang ZB Pei JJ Ma HL Cai PF Yan JK. Effect of extraction media on preliminary characterizations and antioxidant activities of *Phellinus linteus* polysaccharides. *Carbohydr Polym.* (2014) 109:49–55. 10.1016/j.carbpol.2014.03.057 24815400

[31] Jeddou KB Chaari F Maktouf S Nouri-Ellouz O Helbert CB Ghorbel RE. Structural, functional, and antioxidant properties of water-soluble polysaccharides from potatoes peels. *Food Chem.* (2016) 205:97–105. 10.1016/j.foodchem.2016.02.108 27006219

[32] Ji X Cheng Y Tian J Zhang S Jing Y Shi M. Structural characterization of polysaccharide from jujube (*Ziziphus jujuba* Mill.) fruit. *Chem Biol Technol Agric.* (2021) 8:54. 10.1186/s40538-021-00255-2

[33] Nep EI Carnachan SM Ngwuluka NC Kontogiorgos V Morris GA Sims IM Structural characterisation and rheological properties of a polysaccharide from sesame leaves (*Sesamum radiatum* Schumach. & Thonn.). *Carbohydr Polym.* (2016) 152:541–7. 10.1016/j.carbpol.2016.07.036 27516302

[34] Gao J Zhang T Jin ZY Xu XM Wang JH Zha XQ Structural characterisation, physicochemical properties and antioxidant activity of polysaccharide from *Lilium lancifolium* Thunb. *Food Chem.* (2015) 169:430–8. 10.1016/j.foodchem.2014.08.016 25236248

[35] He L Ji P Cheng J Wang Y Qian H Li W Structural characterization and immunostimulatory activity of a novel protein-bound polysaccharide produced by *Hirsutella sinensis* Liu, Guo, Yu & Zeng. *Food Chem.* (2013) 141:946–53. 10.1016/j.foodchem.2013.04.053 23790872

[36] Bruyn AD Anteunis M Gussem RD Dutton GGS. 1H-N.m.r. study of L-rhamnose, methyl cc-L-rhamnopyranoside, and 4-O-β-D-galactopyranosyl-L-rhamnose in deuterium oxide. *Carbohydr Res.* (1976) 47:158–63. 10.1016/s0008-6215(00)83559-4 1268870

[37] Liu J Wen XY Kan J Jin CH. Structural characterization of two water-soluble polysaccharides from black soybean (*Glycine max* (L.) Merr.). *J Agric Food Chem.* (2015) 63:225–34. 10.1021/jf505172m 25494923

[38] Ding HH Cui SW Goff HD Chen J Guo Q Wang Q. Xyloglucans from flaxseed kernel cell wall: structural and conformational characterisation. *Carbohydr Polym.* (2016) 151:538–45. 10.1016/j.carbpol.2016.05.094 27474598

[39] Guo Q Cui SW Wang Q Hu X Wu Y Kang J Structure characterization of high molecular weight heteropolysaccharide isolated from *Artemisia sphaerocephala* Krasch seed. *Carbohydr Polym.* (2011) 86:742–6. 10.1016/j.carbpol.2011.05.018

[40] Luo D. Structural investigation of a polysaccharide (DMB) purified from *Dioscorea nipponica* Makino. *Carbohydr Polym.* (2014) 103:261–6. 10.1016/j.carbpol.2013.12.033 24528728

[41] Xia YG Liang J Yang BY Wang QH Kuang HX. Structural studies of an arabinan from the stems of *Ephedra sinica* by methylation analysis and 1D and 2D NMR spectroscopy. *Carbohydr Polym.* (2015) 121:449–56. 10.1016/j.carbpol.2014.12.058 25659720

[42] Zhao J Zhang F Liu X St Ange K Zhang A Li Q Isolation of a lectin binding rhamnogalacturonan-I containing pectic polysaccharide from pumpkin. *Carbohydr Polym.* (2017) 163:330–6. 10.1016/j.carbpol.2017.01.067 28267513

[43] Xie JH Liu X Shen MY Nie SP Zhang H Li C. Purification, physicochemical characterisation and anticancer activity of a polysaccharide from *Cyclocarya paliurus* leaves. *Food Chem.* (2013) 136:1453–60. 10.1016/j.foodchem.2012.09.078 23194548

[44] Wang L Zhang B Xiao J Huang Q Li C Fu X. Physicochemical, functional, and biological properties of water-soluble polysaccharides from *Rosa roxburghii* Tratt fruit. *Food Chem.* (2018) 249:127–35. 10.1016/j.foodchem.2018.01.011 29407915

[45] Na K Lee KY. Characteristics of the lactan gum produced from various carbon sources by *Rahnella aquatilis*. *Biotechnol Lett.* (1997) 19:1193–5. 10.1023/A:1018429719189

[46] Pohjanlehto H Setälä HM Kiely DE McDonald AG. Lignin-xylaric acid-polyurethane-based polymer network systems: preparation and characterization. *J Appl Polym Sci.* (2014) 131:39714–21. 10.1002/app.39714

[47] Niu Y Shang P Chen L Zhang H Gong L Zhang X. Characterization of a novel alkali-soluble heteropolysaccharide from tetraploid *Gynostemma pentaphyllum* Makino and its potential anti-inflammatory and antioxidant properties. *J Agric Food Chem.* (2014) 62:3783–90. 10.1021/jf500438s 24712394

[48] Wang L Liu HM Qin GY. Structure characterization and antioxidant activity of polysaccharides from Chinese quince seed meal. *Food Chem.* (2017) 234:314–22. 10.1016/j.foodchem.2017.05.002 28551241

[49] Jahanbin K Gohari AR Moini S Emam-Djomeh Z Masi P. Isolation, structural characterization and antioxidant activity of a new water-soluble polysaccharide from *Acanthophyllum bracteatum* roots. *Int J Biol Macromol.* (2011) 49:567–72. 10.1016/j.ijbiomac.2011.06.012 21704651

[50] Liang XX Gao YY Pan Y Zou YF He M He CL Purification, chemical characterization and antioxidant activities of polysaccharides isolated from *Mycena dendrobii*. *Carbohydr Polym.* (2019) 203:45–51. 10.1016/j.carbpol.2018.09.046 30318234

[51] Zeng WC Zhang Z Jia LR. Antioxidant activity and characterization of antioxidant polysaccharides from pine needle (*Cedrus deodara*). *Carbohydr Polym.* (2014) 108:58–64. 10.1016/j.carbpol.2014.03.022 24751247

