# Supplementary material for: Structural Characterization of Polysaccharides in Waste Liquor Produced by Wet Decortication of Sesame Seeds
Source: Front Nutr. 2022 Jun 13;9:940442. doi: 10.3389/fnut.2022.940442 (PMC9234482; doi:10.3389/fnut.2022.940442)
Supplement: Supplementary file 1 [file Data_Sheet_1.docx]

**Supplementary data**

**Structural characterization of polysaccharides in waste liquor produced by wet decortication of sesame seeds**

**Yao-Ran Li^1,2^, Shuai Xu^1^, Run-Yang Zhang^1^, Hua-Min Liu^1*^, Xue-De Wang^1*^**

^1^ *College of Food Science and Technology, Henan University of Technology, Zhengzhou 450001, China*

^2^ *College of Biosystems Engineering and Food Science, Zhejiang University, Hangzhou 310058, China*

*Address correspondence to this author at Henan University of Technology, Zhengzhou, China [E-mail: [liuhuamin5108@163.com](mailto:liuhuamin5108@163.com) (H.M. Liu) and [13903865584@126.com](mailto:13903865584@126.com) (X.D. Wang), Tel: +86-0371-67758021].


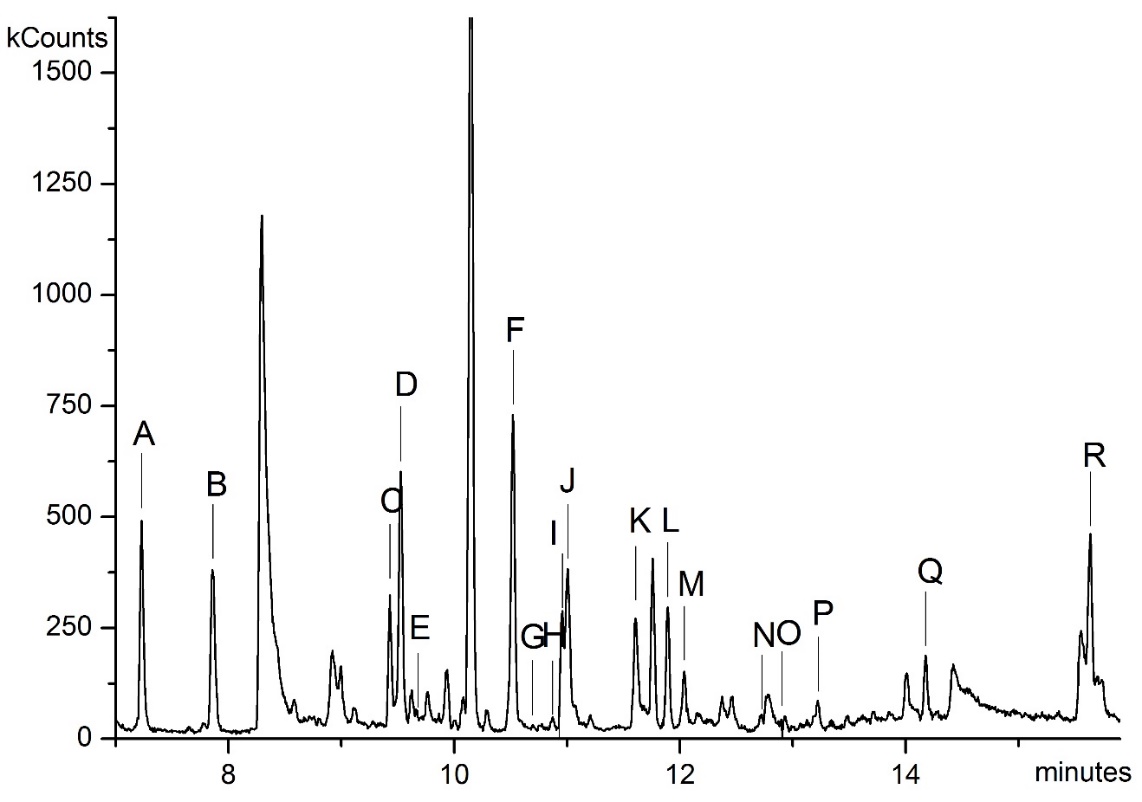


total ion current chromatogram


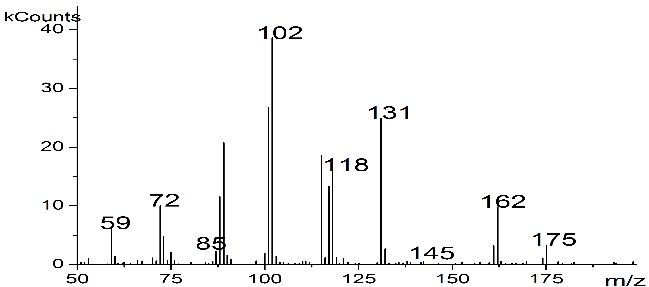

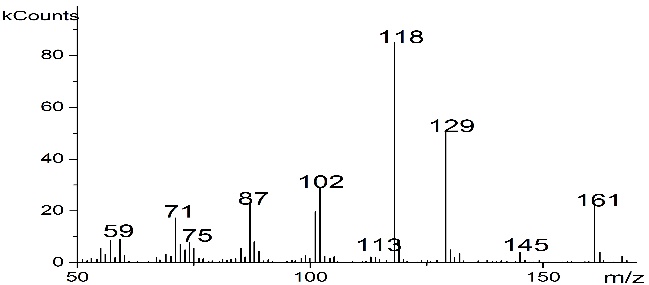
A 2,3,5-Me_3_-Ara*f* B 2,3,4-Me_3_-Rha*p*


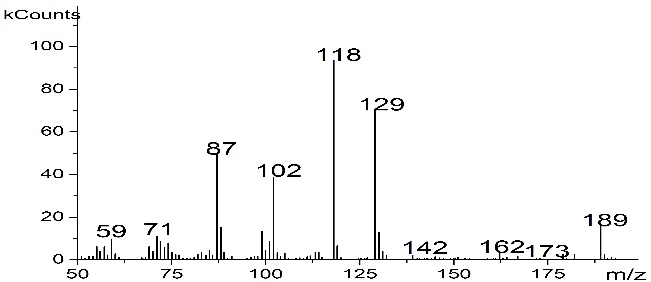

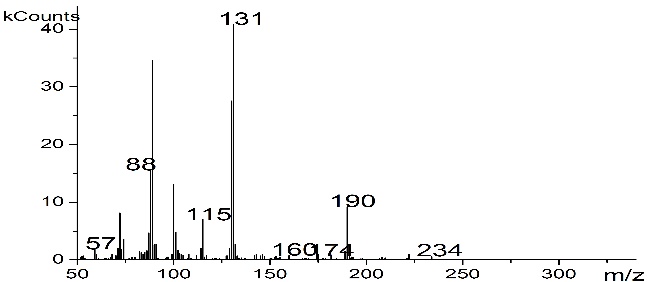
C 3,4-Me_2_-Rha*p* D 2,3-Me_2_-Ara*f*


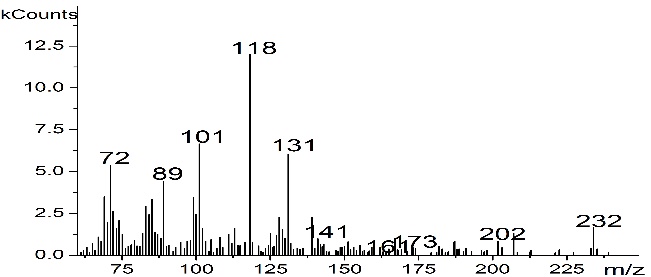

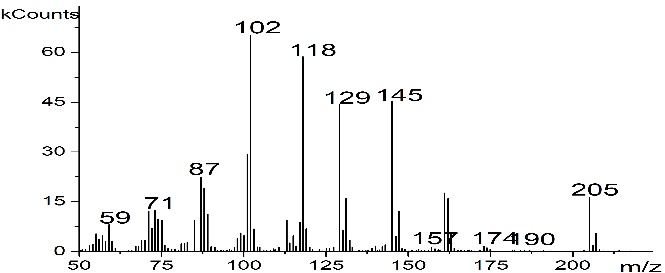
E 2,4-Me_2_-Rha*p* F 2,3,4,6-Me_4_-Gal*p*


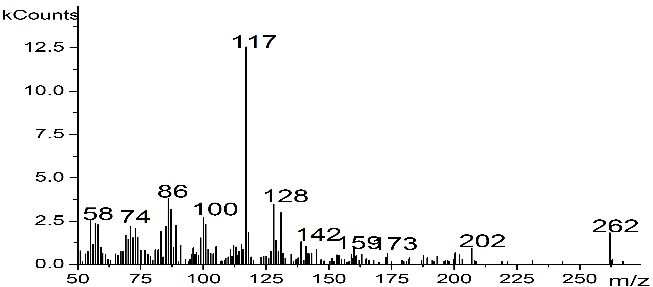

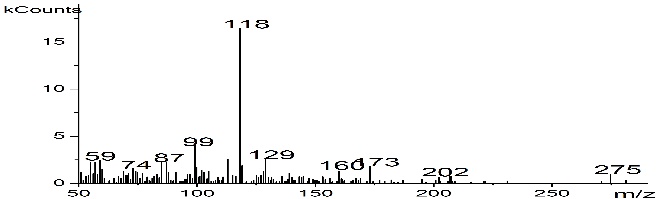
G 2-Me_1_-Rha*p* H 4-Me_1_-Ara*p*


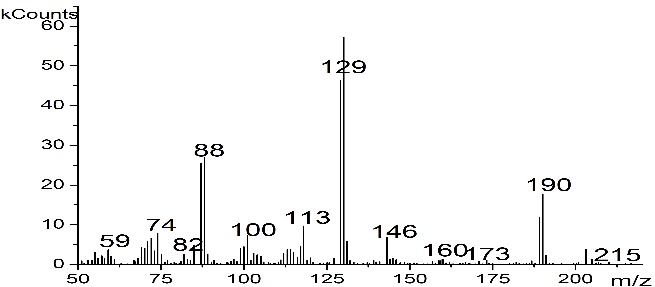

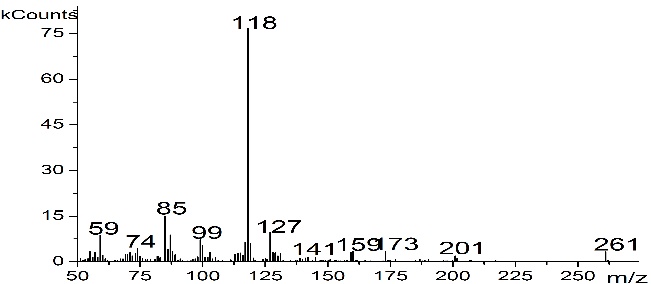
I 2-Me_1_-Ara*f* J 3-Me_1_-Xyl*p*


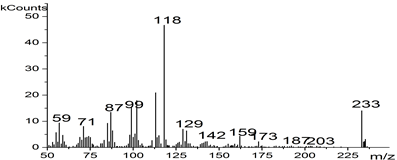

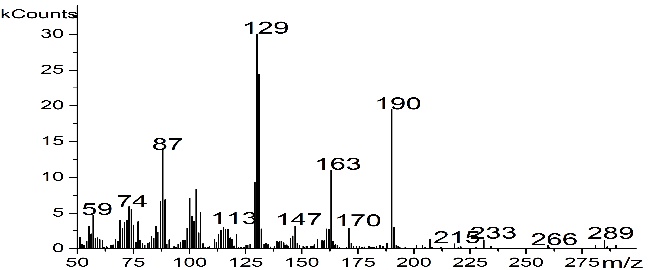
K 3,4,6-Me_3_-Glc*p*A L 2,3,6-Me_3_-Glc*p*


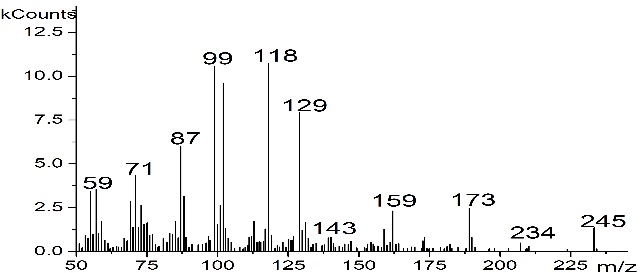

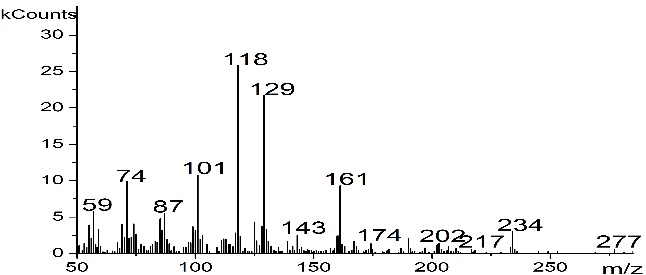
M 2,4,6-Me_3_-Gal*p* N 2,3,4-Me_3_-Gal*p*


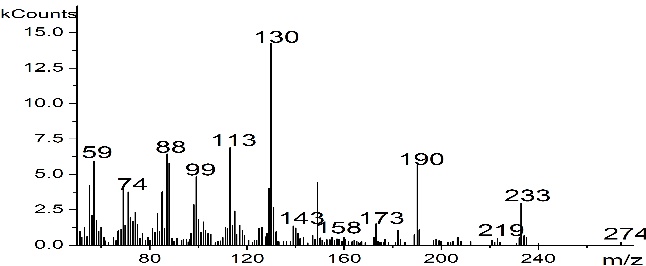

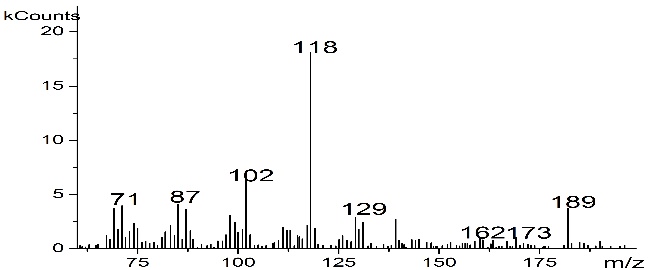
O 2,3-Me_2_-Ara*p* P 3,6-Me_2_-Gal*p*


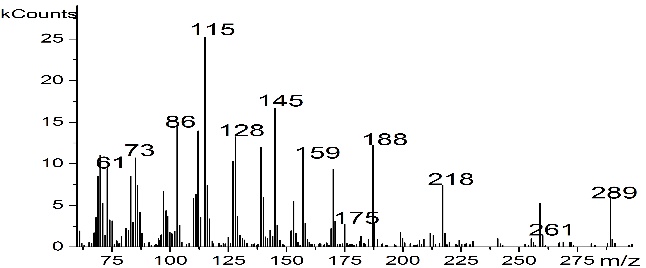

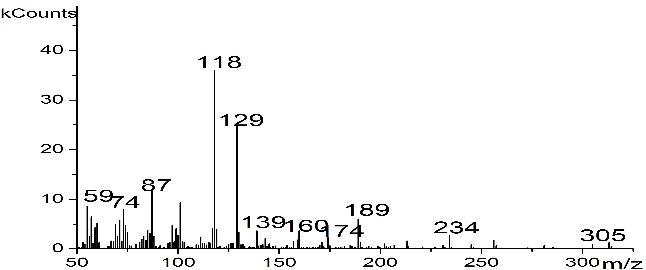
Q 2,4-Me_2_-Glc*p* R Xyl*p*

**Fig. S1.** Linkage analysis: GC-MS chromatograms of the polysaccharide (fraction SSP-2); EI-MS patterns of **PMAA** from fraction SSP-2: (**A**) 2,3,5-Me_3_-Ara*f*; (**B**) 2,3,4-Me_3_-Rha*p*; (**C**) 3,4-Me_2_-Rha*p*; (**D**) 2,3-Me_2_-Ara*f*; (**E**) 2,4-Me_2_-Rha*p*; (**F**) 2,3,4,6-Me_4_-Gal*p*; (**G**) 2-Me_1_-Rha*p*; (**H**) 4-Me_1_-Ara*p*; (**I**) 2-Me_1_-Ara*f*; (**J**) 3-Me_1_-Xyl*p*; (**K**) 3,4,6-Me_3_-Glc*p*A; (**L**) 2,3,6-Me_3_-Glc*p*; (**M**) 2,4,6-Me_3_-Gal*p*; (**N**) 2,3,4-Me_3_-Gal*p*; (**O**) 2,3-Me_2_-Ara*p*; (**P**) 3,6-Me_2_-Gal*p*; (**Q**) 2,4-Me_2_-Glc*p*; (**R**) Xyl*p*.

**Fig. S2.** DTG (A) and TG (B) curves of SSP-1, SSP-2, SSP-3 and SSP-4.
